# Is it a face of a woman or a man? Visual mismatch negativity is sensitive to gender category

**DOI:** 10.3389/fnhum.2013.00532

**Published:** 2013-09-04

**Authors:** Krisztina Kecskés-Kovács, István Sulykos, István Czigler

**Affiliations:** ^1^Experimental Psychology, Research Centre for Natural Sciences, Institute of Cognitive Neuroscience and Psychology, Hungarian Academy of SciencesBudapest, Hungary; ^2^Department of Experimental Psychology, Institute of Psychology, University of DebrecenDebrecen, Hungary; ^3^Department of Cognitive psychology, Institute of Psychology, Eötvös Loránd UniversityBudapest, Hungary

**Keywords:** event-related potential (ERP), gender, perceptual categorization, automatic change detection, facial processing, visual mismatch negativity (vMMN), passive oddball paradigm

## Abstract

The present study investigated whether gender information for human faces was represented by the predictive mechanism indexed by the visual mismatch negativity (vMMN) event-related brain potential (ERP). While participants performed a continuous size-change-detection task, random sequences of cropped faces were presented in the background, in an oddball setting: either various female faces were presented infrequently among various male faces, or vice versa. In Experiment 1 the inter-stimulus-interval (ISI) was 400 ms, while in Experiment 2 the ISI was 2250 ms. The ISI difference had only a small effect on the P1 component, however the subsequent negativity (N1/N170) was larger and more widely distributed at longer ISI, showing different aspects of stimulus processing. As deviant-*minus*-standard ERP difference, a parieto-occipital negativity (vMMN) emerged in the 200–500 ms latency range (~350 ms peak latency in both experiments). We argue that regularity of gender on the photographs is automatically registered, and the violation of the gender category is reflected by the vMMN. In conclusion the results can be interpreted as evidence for the automatic activity of a predictive brain mechanism, in case of an ecologically valid category.

## Introduction

In social interactions of everyday life face recognition is a fundamental function. The human perceptual system can identify categorical attributes of faces, e.g., female, male, happy, fearful, unfamiliar, familiar, etc. Research on face perception concentrated on active, attended paradigms, while contrarily face perception is associated with automatic processes. In our study we were interested in the automaticity of category-formation, more specifically discrimination of female and male faces. On this end we examined this issue using event-related potentials (ERPs), particularly the visual mismatch negativity (vMMN) component. This component is sensitive to registration of environmental regularities and environmental changes even if the visual stimuli are not connected to the attended events, i.e., vMMN is an index of mismatch between the representation of the regularities and the deviant event, without the involvement of attentional processing. VMMN is considered as an error signal to the discrepancy between the expected and actual stimulation (Kimura, 2011; Winkler and Czigler, 2012). VMMN is usually investigated using passive oddball paradigms. In such paradigms, a frequently presented type of stimuli (standard) acquires the representation of regularity, and another, infrequently presented type of stimuli (deviant) violates this regularity. The difference between the ERPs to the deviant and standard is the vMMN component. VMMN is elicited by events physically different from the regular members of stimulus sequences (e.g., color, Czigler et al., 2002; orientation, Astikainen et al., 2008; spatial frequencies, Heslenfeld, 2003; movement, Pazo-Alvarez et al., 2004). Results of a number of studies provide evidence that the sensitivity of vMMN is not restricted to the detection of infrequent changes of elementary features; vMMN is elicited by deviant sequential relationships (Kimura et al., 2011), and the conjunction of visual deviant features (Winkler et al., 2005). Furthermore, it has been shown that the system underlying vMMN is sensitive to perceptual categorization in the color domain (Athanasopoulos et al., 2010; Clifford et al., 2010; Mo et al., 2011), and in Gestalt organization, like vertical symmetry (Kecskés-Kovács et al., 2013), and laterality of human hands as a category (Stefanics and Czigler, 2012).

Before we introduce the main question and procedure of the current study, it is worth to mention some of potentially relevant characteristics of face recognition. The influential functional model of facial processing, developed by Bruce and Young (1986) suggested several face processing units. Among these units the structural encoding module is especially prominent in the context of the present study. This module configures the representation and description of the faces. A well-investigated ERP correlate of the structural encoder is a negative ERP component with 170 ms post-stimulus latency (N170) that reflects the neural mechanisms of face detection (for a review see Bentin et al., 1996).

Concerning the categorical aspects of facial processing, in vMMN studies so far only emotional expressions were investigated (Zhao and Li, 2006; Astikainen and Hietanen, 2009; Stefanics et al., 2012). Zhao and Li (2006), in a modified cross-modal delayed response paradigm where participants performed an acoustic tone discrimination task and ignored the face stimuli (neutral standards vs. happy or sad deviants), obtained vMMN to emotional deviants in an earlier (110–120 ms) and a later (~300 ms) latency range. In this study the negative difference was termed as expression mismatch negativity (EMMN). However, in this experiment the different facial expressions were produced by a single actor (the various emotions were produced by only one person). Therefore, it is possible that the ERP difference was due to low-level feature changes. Similarly, in another study (Susac et al., 2004) the facial emotions were expressed by a single actor. The possibility of low-level visual effects was eliminated by Astikainen and Hietanen (2009). In their study emotions were presented by different actors. In this study the passive oddball paradigm and the task-related events were in the auditory modality. The ERPs to the deviant face stimuli (happy vs. fearful) were more negative in an earlier (140–160 ms) and in a later (280–320 ms) latency range. Astikainen et al. regarded the second vMMN as the relevant index of emotional change detection. The authors suggested that in an earlier latency range (140–160 ms) deviant-related negativity was a consequence of the change of the face-related N170 ERP component.

In a passive oddball paradigm Stefanics et al. (2012) introduced a visual detection task at the center of the visual field with a fixation cross. The vMMN-related face stimuli were presented parafoveally. They observed deviant-related negativities in two latency ranges (150–220 and 250–360 ms). Furthermore, they found different hemispheric lateralization for the positive and negative automatic emotional processes (fearful-right, happy-left hemisphere).

In the majority of studies face identity varied within sequences, consequently at the level of elementary visual features different cell populations were stimulated. Therefore, stimulus variability decreased the possibility of stimulus-specific refractoriness of exogenous activity (May and Tiitinen, 2010). However, the similar latency (Astikainen and Hietanen, 2009) and the decreased effect in an equal probability control (Li et al., 2012) indicates, refractoriness of higher order processing structures, specific to face processing may be involved in the early sub-component of emotion-related vMMN^1^. However, the later negative effect (in the 250–360 ms range) seems to be a valid genuine vMMN. On the basis of the results of vMMN studies with facial stimuli we expected a similar latency range for the gender-related deviant-*minus*-standard difference potentials. This vMMN component would reflect the higher level of the automatic change detection that is related to facial gender categories.

Gender-related categorical perception was investigated less frequently than the emotional categories. In their behavioral study, Campanella et al. (2001) examined the processes of gender perception in a delayed matching task with morphed unfamiliar face pairs. They found a morphing main effect (the participants identified gender easily if the distance of morph was large between pictures). Additionally, and more interestingly, it was easier to discriminate between-gender pairs than within-gender pairs, even if the morphing differences were identical.

In a second relevant study Mouchetant-Rostaing et al. (2000) recorded three types of gender-processing. In the first condition all faces were identical gender (female vs. male—preventing gender discrimination). In the second condition, both types of gender were presented, but gender itself was irrelevant for the participant's task. The third condition was an explicit gender discrimination task. The main finding was that gender processes are different from the structural encoding of faces (N170). Gender categorization effect was observed (in the second and third conditions) in the later epoch range (200–250 ms) which might reflect more general gender categorization processes. As results of ERP data suggests, it seems that representation and encoding of gender information on the face is automatic.

In the present study, we investigated whether gender category was capable of eliciting vMMN, when male faces as deviants were presented in a sequence of female faces and *vice versa*.

## Experiment 1

### Materials and methods

#### Participants

Participants were 14 healthy adults [6 women; mean age = 21.16 years, standard deviation (*SD*) = 1.52 years]. They had normal or corrected-to-normal vision. Written informed consent was obtained from every participant before the experimental procedure. The study was conducted in accordance with the Declaration of Helsinki, and accepted by the United Committee of Ethics of the Psychology Institutes in Hungary.

#### Stimuli

The stimuli were 80 cropped faces with neutral expression, 40 from each gender taken from public internet databases (from internet database: www.findaface.ch, we attempted to avoid pictures with emotional experiences, i.e., the photographs were “college yearbook” types). Using Photoshop (CS4) software, black and white pictures were created with a specific cropping (the size of the cropping mask was 1024 × 1024 pixels i.e., 12.9°)^2^.

**Figure 1:**
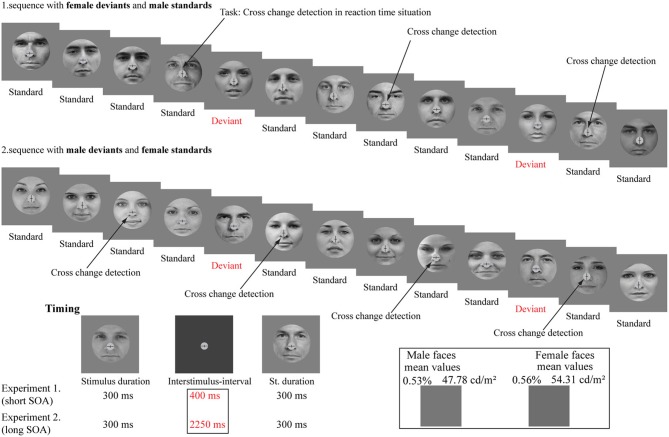
**Stimuli and illustration of the sequence applied**. The schematic illustration shows the presented pattern of the gender faces and the cross-change detection task. The stimuli were photographs of eighty different persons (40 females and 40 males). The lower left panel shows the experimental timing. The lower right panel shows the mean luminance (cd/m^2^) of the stimuli and the mean grayscale values (%; pixel-by-pixel mean values).

Stimulus duration was 300 ms and the inter-stimulus-interval (ISI; i.e., non-stimulated interval) was 400 ms (see Figure 1).

The background was gray (36.67 cd/m^2^). The mean luminance of female faces was 54.31 cd/m^2^ (*SE* = 2.0 cd/m^2^). Male faces were presented with 47.78 cd/m^2^ mean luminance value (*SE* = 2.0 cd/m^2^). Stimuli appeared on a 17” monitor (Samsung SyncMaster 740B, 60-Hz refresh rate) from a 1.2 m viewing distance in a dimly lit and soundproof room.

The probability of frequent stimuli (standard) was 0.8% and the probability of infrequent stimuli (deviant) was 0.2%. We applied two conditions (female deviant and male deviant). In one of the conditions female faces were the frequent (standard) and male faces were the infrequent (deviant) stimuli. In the other condition these probabilities were reversed. There were 600 stimuli (480 standards and 120 deviants) in a condition. The order of presentation of conditions was counterbalanced across participants. The number of consecutive standards was changed in pseudo random order from two to nine. The successive stimuli were never physically identical.

#### Task

Participants performed a simple reaction time (RT) task. The center of the screen was the task-field, which included a gray circle (0.81° with 36.67 cd/m^2^). The target was a dark cross (0.45 cd/m^2^), continuously presented at the center of the circle. The participants were instructed to detect the change of dark cross (the cross changed random between 5 and 15 s). The cross comprised of a shorter (0.37°) and a longer line (0.75°), and responses were required for each reversal of the size of the lines. Central fixation was required, and participants were asked to respond as quickly and as correctly as possible. The participants responded to the changes by pressing a button.

#### EEG measuring

EEG was recorded (DC-30 Hz, sampling rate 500 Hz; Synamps2 amplifier, NeuroScan recording system) with Ag/AgCl electrodes placed at 61 locations according to the extended 10–20 system using an elastic electrode cap (EasyCap). The right mastoid was used as reference, off-line re-referenced to average activity. Horizontal EOG was recorded with a bipolar configuration between electrodes positioned lateral to the outer canthi of the two eyes. Vertical eye movements were monitored with a bipolar montage between electrodes placed above and below the right eye. The EEG signal was band pass filtered offline, with cutoff frequencies of 0.1 and 30 Hz (24 dB slope). Epochs of 800 ms duration (including a 100 ms pre-stimulus interval) were extracted for each event and averaged separately for standard and deviant stimuli (from the two conditions female and male deviants). The mean voltage during the 100 ms pre-stimulus interval was used as the baseline for amplitude measurements, and epochs with an amplitude change exceeding ±70 μ V on any channel were rejected from further analysis.

Only responses from the third to ninth standard after a deviant were included in the standard-related ERPs. To identify change-related activities, ERPs from standard stimuli were subtracted from ERPs from deviant stimuli of the respective condition.

#### Analyses and comparisons

As the results of the majority of vMMN studies suggest, we expected the emergence of deviant-*minus*-standard difference over the posterior electrode locations. However, to reinforce this expectation, we defined a channel matrix on the basis of results of point-by-point *t*-tests (criterion: minimum 10 consecutive significant data points, i.e., 20 ms; see e.g., Guthrie and Buchwald, 1991) applied on the whole scalp location. The largest significant difference (deviant-*minus*-standard) appeared on the matrix of ten electrodes (P7, PO3, POz, PO4, P8, PO7, O1, Oz, O2, and PO8). This matrix consisted of two rows (factor of anteriority: anterior and posterior) and five columns (factor of laterality: left, left-middle, middle, right-middle, and right).

On the basis of previous face-related vMMN (e.g., Stefanics et al., 2012; face stimuli elicited vMMN-related negativity in 150–360 ms latency range) difference potentials as vMMN were identified from grand-average waveforms in the 202–498 ms range. In vMMN-related analyses of variance (ANOVAs) the mean amplitude values of this epoch were used.

Three-Way ANOVAs were introduced with factors of *Stimulus Type* (standard and deviant), *Anteriority* (anterior and posterior), and *Laterality* (left, left-middle, middle, right-middle, and right). Amplitude and peak latency values of the P1 and N1/N170 components were analyzed in similar ANOVAs. However, faces elicit a more negative response at lateral occipital electrode locations, especially over the right hemisphere (especially PO8 electrode and PO7, P7, P8; see Bentin et al., 1996, for a review).

When appropriate, Greenhouse-Geisser correction of the degrees of freedom was applied and the ε values are reported in the results. Significant effect's sizes were represented by the partial eta-squared. Furthermore, significant interactions were further specified by Tukey HSD *post-hoc* tests. Surface distributions were compared under method of the vector-scaled amplitude values (McCarthy and Wood, 1985). Additionally, we calculated the mean amplitude value of two exogenous components (P1 and N1/N170) in a ±20 ms epoch around the peak amplitude value of the group average. Moreover, rare deviant responses included both types (female and male) of visual events violating sequential regularities.

### Results

#### Behavioral results

The participants performed the primary task with hit rates over 80% (mean hit rate = 95.30%, *SD* = 5.18%). The median RT was 485.5 ms (*SD* = 125.00 ms). There was no difference in performance between the conditions.

#### Event-related potentials

**Figure 2:**
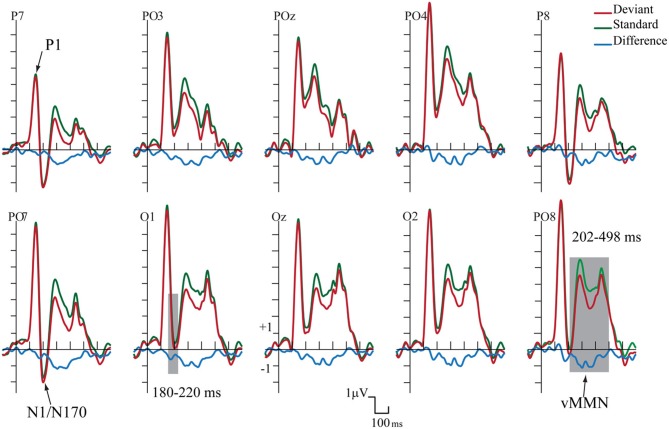
**Group average event-related potentials for frequent (standard), for infrequent (deviant) stimuli, and their difference potential**. Shaded areas mark the intervals where significant differences were largest. The early (180–220 ms) negative difference is considered as an amplitude modulation of the N1/N170 component. Deviants elicited negativity in 202–498 ms latency range that is sensitive to gender categorization processes.

**Figure 3:**
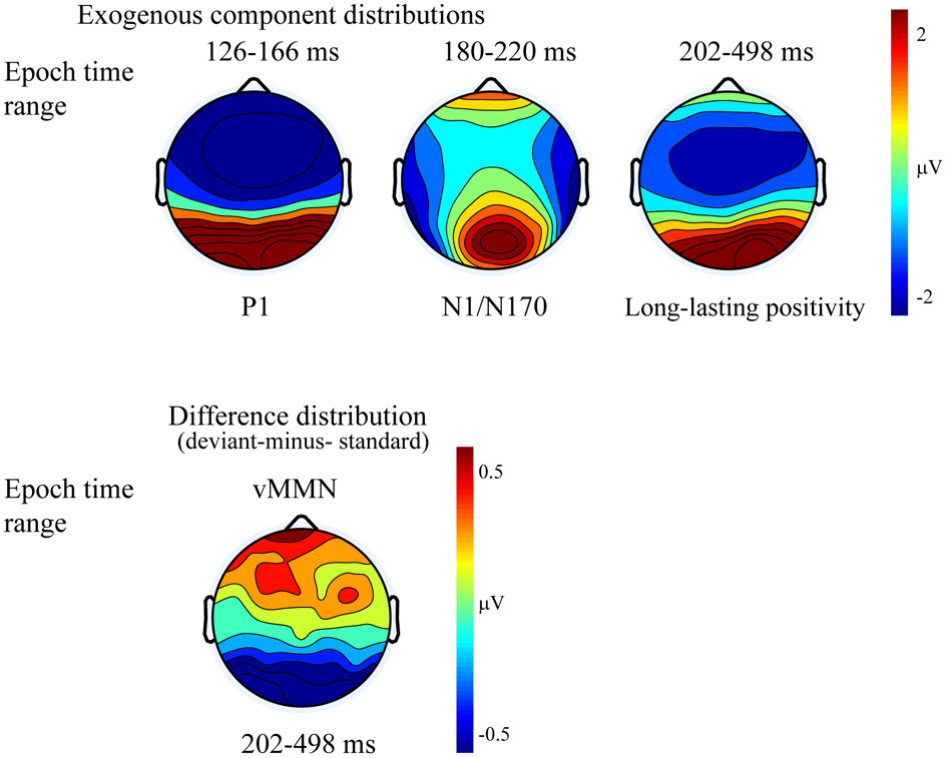
**Topographic maps (surface distribution) of the exogenous components (P1, N1/N170, Long-lasting positivity) and the deviant-minus-standard difference potential in the 202–498 ms range**. Color represents the amplitude values.

Figure 2 shows the ERPs to deviant and standard stimuli, and the deviant-*minus*-standard difference potentials. As Figure 2 shows, stimuli elicited a large positive component within the 126–166 ms range (P1) with amplitude maximum at the PO8 channel location (146 ms). We obtained no P1 amplitude and latency difference for frequent and infrequent stimuli. The P1 was followed by a small negative component in the 180–220 ms latency range (N1/N170), and a long-lasting positivity in the 202–498 ms range. Figure 3 (upper panel) shows the topographic maps of exogenous components to standard stimuli and the surface distribution of the deviant-*minus*-standard difference potentials in the 202–498 ms range. Furthermore, Table 1 shows the peak amplitude values of the P1 and N1/N170 components and the largest negative values of the difference potentials.

**Table 1:** **Mean amplitude values (μV) and mean latency values (ms) of the event-related potentials to standard and deviant face stimuli (Standard error of the mean in parenthesis)**.

	**P1 126–166 ms**		**N170 180–220 ms**		**vMMN 202–498 ms**
	**Standard**	**Deviant**	**Standard**	**Deviant**	**Deviant-*minus*-Standard**
PO7 Amplitude values	6.75 (0.48)	6.52 (0.41)	−1.17 (1.35)	−1.53 (1.33)	−0.76 (0.15)
PO7 Latency values	146 (1.49)	145 (1.43)	205 (5.22)	209 (5.49)	347 (6.95)
PO8 Amplitude values	8.06 (0.81)	8.06 (0.82)	0.67 (1.12)	0.19 (1.20)	−0.63 (0.18)
PO8 Latency values	146 (1.09)	145 (1.83)	203 (4.14)	202 (4.30)	358 (6.44)

As Figure 2 shows in the 202–498 ms latency range the ERP to deviants was more negative than the ERP to standards.

On the basis of *t*-tests we obtained significant deviant-standard difference within the 160–498 ms latency range. Due to the similarity of the earlier part of this range to the latency range of a negative epoch of the ERPs (N1/N170) and the dissimilarity of the later deviant-related negativity to the long-lasting ERP positivity, we conducted separate ANOVAs for earlier and later effects.

As for the P1 component, amplitudes (mean of the 126–166 ms range) were compared to deviant and standard stimuli. We obtained no significant difference. However, in the N1/N170 range (180–220 ms) the negativity was larger to deviant stimuli than standards. In the Three-Way ANOVA *Stimulus Type* and *Laterality* main effects were significant [*F*_(1, 13)_ = 9.75, *p* < 0.01, η^2^ = 0.42 and *F*_(4, 52)_ = 14.84, *p* < 0.001, η^2^ = 0.53, ε = 0.48, respectively]. These significant effects indicate larger negative responses to deviant compared to standard gender face stimuli, and both type of stimuli elicited larger response at the lateral electrode locations than in the midline (PO7, P7 and PO8, P8). Finally, *Anteriority X Laterality* interaction [*F*_(4, 52)_ = 14.03, *p* < 0.001, η^2^ = 0.51, ε = 0.48] was due to the larger negativity over the posterior locations in the extreme lateral locations. The latencies of P1 to standard and deviant stimuli were almost identical within the electrode matrix.

It is possible that, instead of the emergence of early memory mismatch effect (vMMN; Zhao and Li, 2006; Stefanics et al., 2012), the deviant-related negativity effect is an amplitude modulation of the N1/N170 component. For that reason we compared the surface distribution of the N1/N170 to the standard stimuli and the distribution of early difference potential (deviant-*minus*-standard difference). On this end vector-scaled amplitude values (McCarthy and Wood, 1985) were used in an ANOVA with factors of *Component* (standard and difference potential), *Anteriority* and *Laterality*. In this analysis there were neither significant main effects of component [*F*_(1, 13)_ = 0.22, *p* = 0.64, η^2^ = 0.01] nor interactions. Accordingly, we obtained no evidence of genuine mismatch activity in the earlier latency range, i.e., the early difference is an increased amplitude value of the N1/N170 component.

The deviant-related activity (vMMN) was analyzed in the 202–498 ms latency range. All main effects were significant, *Stimulus Type* main effect [*F*_(1, 13)_ = 18.83, *p* < 0.001, η^2^ = 0.59]; *Anteriority* main effect [*F*_(1, 13)_ = 14.86, *p* < 0.01, η^2^ = 0.53]; and *Laterality* main effect [*F*_(4, 52)_ = 43.84, *p* < 0.001, η^2^ = 0.50, ε = 0.59, respectively]. We consider the significant difference between deviant and standard as vMMN component. Furthermore, the interaction of *Anteriority* and *Laterality* was also significant [*F*_(4, 52)_ = 11.09, *p* < 0.001, η^2^ = 0.46, ε = 0.73], showing that ERPs were larger over the posterior, extreme right locations.

### Discussion

Experiment 1 demonstrated that the face stimuli elicited two ERP components in the earlier latency range (up to 220 ms). The large P1 component was insensitive to the probability of genders. Faces elicited an N1/N170 component, however the negativity had small amplitude. More importantly, we recorded more negative responses to rare (deviant) stimuli than to standard stimuli although this difference (deviant-*minus*-standard) can be an amplitude modulation of the N1/N170 component.

The ISI of Experiment 1 was shorter (400 ms) than the ISI of the typical studies of reported fairly large N170. The short ISI might contribute to the attenuated exogenous activity (e.g., Czigler, 1979; Liu et al., 2010). On the one hand, considering the N170 component as an index of the structural encoding of faces (c.f. Bentin et al., 1996), it is possible that the repeated presentation of the structural features characteristic to a gender (male or female) may saturate the processes underlying this component, therefore, as mentioned above, the early deviant-*minus*-standard difference effect was a manifestation of the refractoriness of the face-specific activity. On the other hand, in this experiment genders were effectively discriminated, and considering that structural encoding is a necessary stage of such discrimination, (N170 component is an index of structural encoding processes), it seems that the amplitude of the N170 component is unrelated to successful encoding. As an alternative, there is no close connection between the processes underlying the N170 component and the encoding processes necessary for gender discrimination^3^.

The main finding of this experiment is the long-lasting deviant-related posterior negativity to facial stimuli of the infrequent gender. Emergence of this deviant-related negativity in the later latency range cannot be explained as a refractoriness effect, because in this range exogenous activity was mainly positive. Refractoriness of positive ERP components to standard, and the lack of refractoriness to deviant stimuli should result in positive, instead of negative difference potential. Therefore, the posterior negativity of the 202–498 ms range is considered as a vMMN, elicited by the ecologically significant change of gender category. The findings of Experiment 1 provided further evidence that the violation of the rule: “members of a particular category are presented sequentially,” automatically registered in the perceptual system.

In summary, in the present Experiment, due to the short ISI the face-related exogenous activity was unexpectedly small. In Experiment 2 we introduced longer ISI. Besides the possibility of an enlarged N1/N170 component, we investigated the ISI-effect on the vMNN component. Because this component is considered to depend on the short-term registration of sequential rules (Astikainen et al., 2008), we expect an enlarged N1/N170 and the reduction of vMMN amplitude in Experiment 2.

## Experiment 2

### Materials and methods

#### Participants

Participants were 12 healthy adults (3 women; mean age = 21.50 years, *SD* = 1.78 years). They had normal or corrected-to-normal vision. Written informed consent was obtained from every participant before the experimental procedure. The study was conducted according to the Declaration of Helsinki, and accepted by the United Committee of Ethics of the Psychology Institutes in Hungary.

#### Stimuli, procedure, EEG measuring, and data processing

Experiment 2 was almost identical to Experiment 1. We manipulated only the ISI that was between 2000 and 2500 ms (mean ISI: 2250 ms; we calculated with a pseudorandom value drawn from the standard uniform distribution on the interval). The other stimulus parameters, the participant's task, EEG recording (except the online filter: DC-100 Hz) and data processing were the same as Experiment 1.

### Results

#### Behavioral results

All participants performed the primary task with hit rates over 80% [mean hit rate = 97.84% (*SD* = 5.56%)]. The median RT was 463.38 ms (*SD* = 123.16 ms). There was no difference in performance between the conditions.

#### Event-related potential data

**Figure 4:**
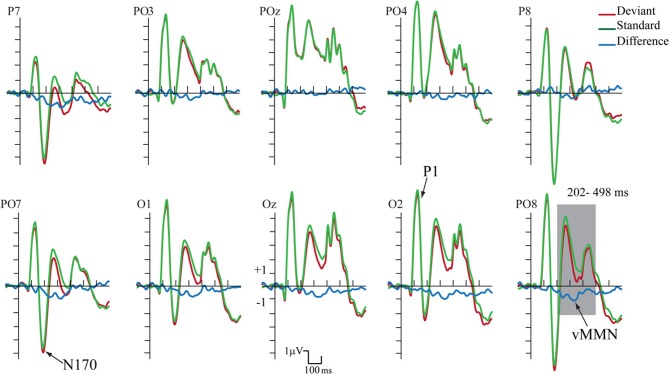
**ERP's to standards and deviants stimuli and their difference potential (in case of the longer ISI)**. Contrary to Experiment 1 no N1/N170 amplitude difference appeared to deviants and standards. However, the infrequent deviant faces elicited enhanced negativity in the 202–498 ms latency range, i.e., a category-related vMMN component (shaded area represents the largest difference).

**Figure 5:**
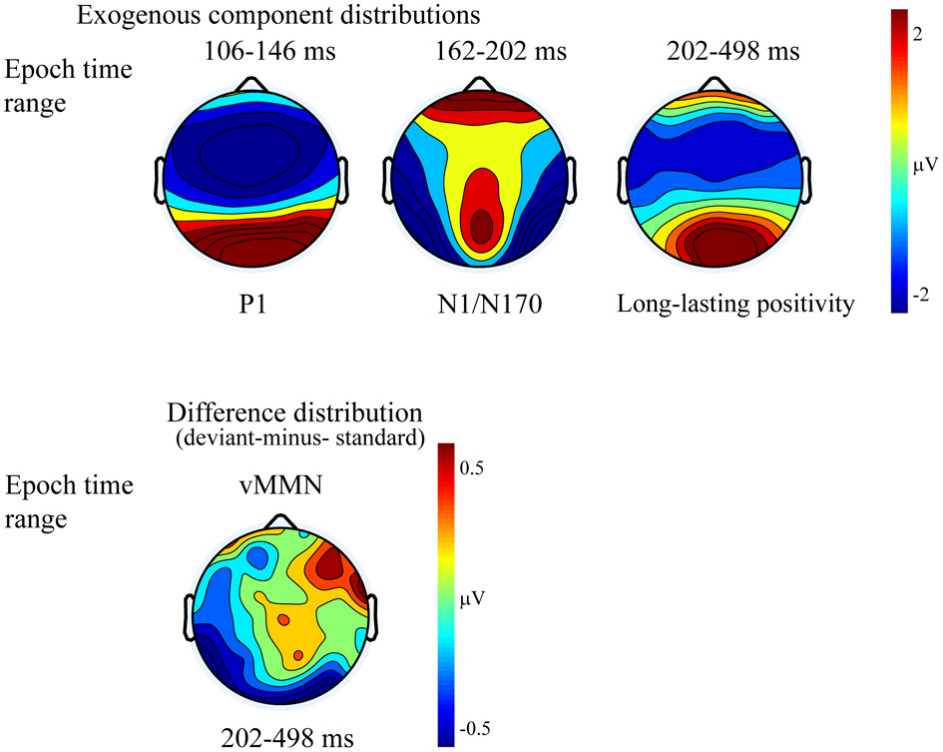
**Topographic maps (surface distribution) of the exogenous components (P1, N1/N170, long-lasting positivity) and the deviant-minus-standard difference potential in the 202–498 ms range**.

Figure 4 shows the ERPs to deviant and to standard stimuli, and the deviant-*minus*-standard difference potential. As the figure shows, in this experiment the P1 component was followed by a large N1/N170 component. The other aspects of the ERPs were similar to the ERPs in Experiment 1, since deviant stimuli elicited a long-lasting negativity within the 202–498 ms latency range. Figure 5 shows the surface distribution of the exogenous components and the difference potential (in construction identical to that of Figure 3), and Table 2 shows the amplitude values of the exogenous components and the difference potential.

**Table 2:** **Mean amplitude values (μV) and mean latency values (ms) of the event-related potentials to standard and deviant face stimuli (Standard error of the mean in parenthesis)**.

	**P1 106–146 ms**		**N170 162–202 ms**		**vMMN 202–498 ms**
	**Standard**	**Deviant**	**Standard**	**Deviant**	**Deviant-*minus*-Standard**
PO7 Amplitude values	4.37 (0.46)	3.94 (0.47)	−3.97 (0.86)	−4.24 (0.29)	−0.50 (0.25)
PO7 Latency values	129 (3.29)	124 (3.85)	189 (3.84)	190 (4.15)	345 (5.58)
PO8 Amplitude values	6.04 (1.37)	5.71 (1.40)	−5.26 (0.83)	−5.62 (1.42)	−0.63 (0.11)
PO8 Latency values	123 (3.05)	124 (2.83)	183 (3.62)	185 (3.68)	344 (3.64)

The P1 had a wide posterior distribution, while the N1/N170 component emerged over the bilateral posterior locations. The difference potential had a restricted distribution over the posterior locations.

We applied the same statistical analyses as in Experiment.1. The deviant-*minus*-standard difference potential was statistically significant within the 120–480 ms latency range.

The P1 component had maximum on the PO8 channel location (126 ms). We obtained no significant *Stimulus Type* effect on this component. However, *Anteriority* [*F*_(1, 11)_ = 12.12, *p* < 0.01, η^2^ = 0.52] and *Laterality* [*F*_(4, 44)_ = 5.92, *p* < 0.001, η^2^ = 0.35, ε = 0.40] main effects were significant. According to the Tukey HSD tests, the P1 component was larger at the posterior row and the P1 amplitude was larger at the midline locations (*p* < 0.01 in all cases). As for latency values, in a similar ANOVA no significant effect appeared.

Unlike in Experiment 1, we obtained no significant N1/N170 amplitude difference between the ERPs to deviants and standards. In the Three-Way ANOVA the bilateral maxima of this component is reflected by the significant *Laterality* main effect [*F*_(4, 44)_ = 22.30, *p* < 0.001, η^2^ = 0.67, ε = 0.50]. In addition, *Anteriority* main effect was also significant: [*F*_(1, 11)_ = 9.96, *p* < 0.01, η^2^ = 0.47]. According to the Tukey HSD test, the N1/N170 component had larger negative values at the bilateral posterior locations (at the P7, PO7 and P8, PO8 channels *P* < 0.01 in all cases). *Anteriority × Laterality* interaction was also significant [*F*_(4, 44)_ = 12.45, *p* < 0.001, η^2^ = 0.53, ε = 0.50]; this effect was due to the more negative values (for both deviants and standards) at the posterior and lateral locations. In ANOVAs on the N1/N170 latency values there were neither significant main effects nor interactions.

In the 202–498 ms latency range the Three-Way ANOVA resulted in significant main effects of *Stimulus Type* [*F*_(1, 11)_ = 7.40, *p* < 0.01, η^2^ = 0.40] and *Laterality* [*F*_(4, 44)_ = 17.37, *p* < 0.001, η^2^ = 0.61, ε = 0.57]. *Stimulus Type* main effect indicated enhanced negativity to the changes of gender category (vMMN), even if the ISI increased to 2250 ms. Besides, the *Laterality* main effect showed that the vMMN maxima was located at the PO7 and PO8 channel locations. Finally, significant *Stimulus Type × Anteriority* interaction [*F*_(1, 11)_ = 7.00, *p* < 0.05, η^2^ = 0.38] reflected larger vMMN at the posterior locations. Latency values of the late long-lasting components to standards and deviants were not different.

### Discussion

The vMMN effect of Experiment 2 replicated the results of Experiment 1, even if the ISI was longer. The amplitude of the exogenous activity (N1/N170) increased at longer ISI. As a plausible explanation, at longer ISI the refractory effect on this component dissipated, and the lack of deviant-related N1/N170 difference was due to the saturation of the amplitude, even in the case of standard stimuli.

## Comparison of the first and second experiment's results

**Figure 6:**
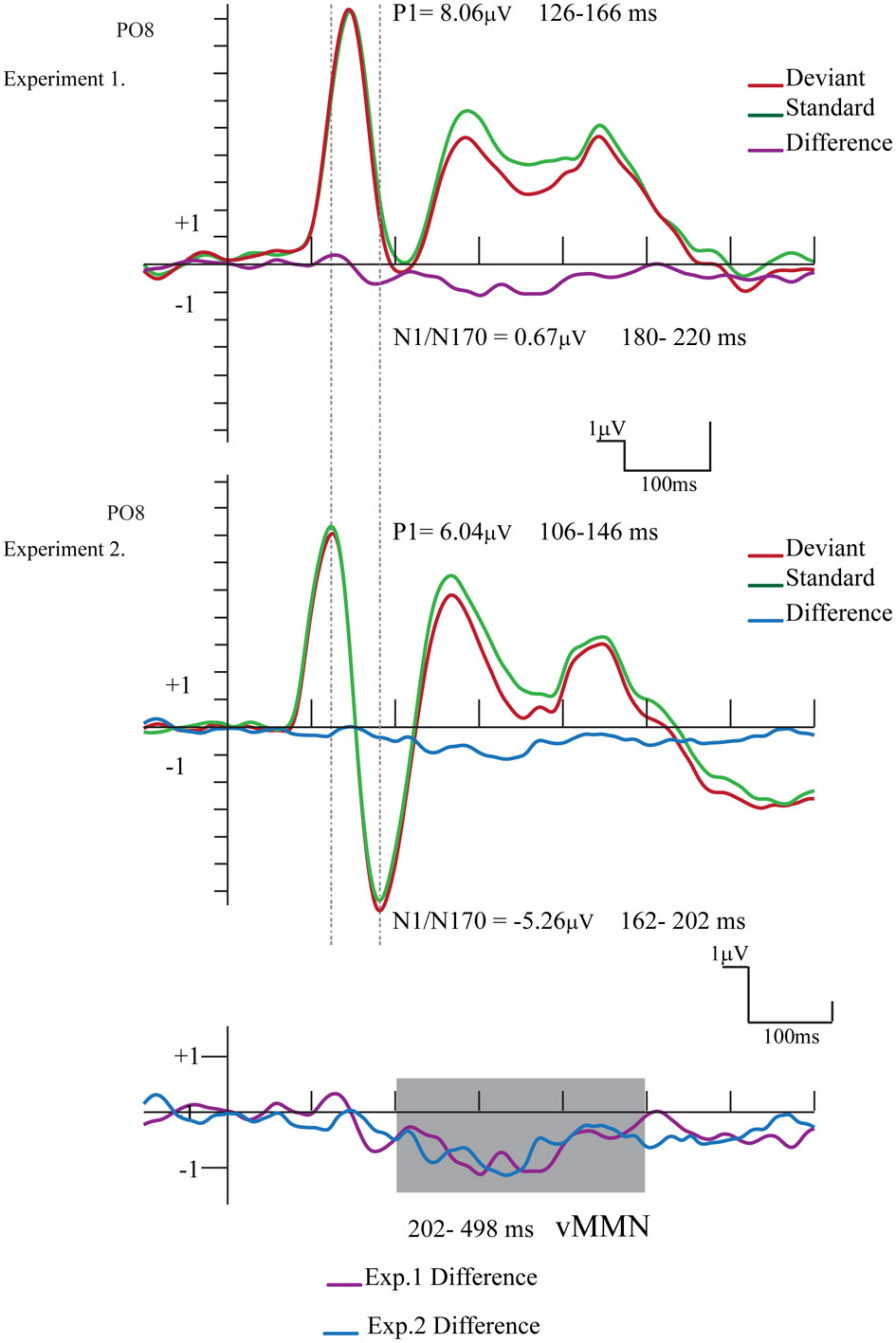
**Comparison of the ERPs and difference potentials of Experiment 1 and Experiment 2**. The epoch range and mean amplitude values are presented (the shaded area marks the latency range of negative differences). We found amplitude and latency differences between the exogenous components.

Figure 6 compares the ERPs and difference potentials of Experiment 1 (short ISI) and Experiment 2 (long ISI) at the PO8 channel location. The figure illustrates three obvious differences: the latency of P1 and N1/N170 components were longer in Experiment 1, and the N1/N170 amplitude was more negative in Experiment 2. As a less evident difference, the P1 amplitude was larger in Experiment 1.

The P1 amplitude difference was investigated in an ANOVA with factors of *Experiment* (short ISI in Experiment 1 vs. long ISI in Experiment 2; between group factors) and *Laterality* (PO7 and PO8 channels). *Experiment* main effect was significant [*F*_(1, 24)_ = 5.16, *p* < 0.05, η^2^ = 0.17], showing that the amplitude values of the P1 component was really larger in Experiment 1.

As for the latency difference, in a similar ANOVA the main effect of *Experiment* was significant [*F*_(1, 24)_ = 39.56, *p* < 0.001, η^2^ = 0.62], indicating that the latency was shorter in Experiment 2. Furthermore, for the latency values the significant *Experiment × Laterality* interaction [*F*_(1, 24)_ = 4.64, *p* < 0.05, η^2^ = 0.16] shows that in Experiment 2 the P1 latency was longer over the right (PO8) side.

The N1/N170 amplitudes and latencies were analyzed in similar ANOVAs. The amplitude values of the N1/N170 component were different between the two experiments [*F*_(1, 24)_ = 8.61, *p* < 0.01, η^2^ = 0.26]. Furthermore, the difference was larger over the right side, as indicated by the significant *Experiment* × *Laterality* interaction [*F*_(1, 24)_ = 10.85, *p* < 0.005, η^2^ = 0.31]. Latency values of the N1/N170 component were also significantly different in the two experiments [*F*_(1, 24)_ = 10.74, *p* < 0.01, η^2^ = 0.30], showing a shorter latency in case of longer ISI (Experiment 2).

Finally, we compared the vMMN scalps distributions (202–498 ms) in the two experiments at a 2 × 5 electrode matrix (P7, PO3, POz, PO4, P8, PO7, O1, Oz, O2, PO8). In this ANOVA we used vector-scaled amplitude values (McCarthy and Wood, 1985). In ANOVA the main effect of *Experiment* and the interactions (*Experiment × Anteriority; Experiment × Laterality; Experiment × Anteriority × Laterality*) were not significant i.e., we obtained no significant difference between the vMMN distributions.

## General discussion

Concerning the exogenous components, the P1 component had larger amplitude in Experiment 1 than in Experiment 2, (8.06 vs. 6.04 μ V), therefore there was no ISI (400 vs. longer 2250 ms) and so there was no refractory effect on P1. In general, the processes of refractoriness attributed to the decreased responsiveness of neurons for the “fast” repeated input (see Näätänen and Picton, 1987, for a review). Therefore, in the case of the longer interval between the successive stimuli, larger exogenous components should have occurred. However, the characteristic of the exogenous P1 component (i.e., larger amplitude and latency) did not follow the prediction based on the refractory theory.

On the contrary, at longer ISI (2250 ms) the human face stimuli elicited an enlarged N1/N170 component (≤5.26μ V at PO8). The posterior bilateral distribution of the negativity corresponded to the findings reporting the face-related N170 component (e.g., Bentin et al., 1996). In case of short ISI (Experiment 1) we obtained a small deviant-related difference on N1/N170-effect of infrequent deviant gender stimuli were embedded in a sequence of frequent patterns. However, this difference disappeared at longer ISI (Experimental 2). Therefore, the small early amplitude difference, as a N1/N170 modulation in Experiment 1 has to be treated carefully. Nevertheless, category specific refractoriness is a possible explanation, although the long ISI of the present study is beyond the interval which is sensitive to refractoriness effect (Coch et al., 2005).

In both experiments facial stimuli belonging to the infrequent gender category of a sequence elicited vMMN. The results of the present study are in line with the behavioral results of Campanella et al. (2002) showing the sensibility of the perceptual system to gender as category. VMMN in this study emerged as a long lasting ERP component with the onset of ~200 ms post stimulus, and terminated at ~500 ms. The onset time corresponds to the results of other vMMNs studies, where another facial category, emotional expression established the sequential regularity (Zhao and Li, 2006; Astikainen and Hietanen, 2009; Li et al., 2012; Stefanics et al., 2012). The results of Susac et al. (2004) also supported the possibility of late vMMN to facial category change without an amplitude difference in an earlier (N1/N170) latency range.

In comparison to the emotion-related vMMN, in the present study the duration of the vMMN component was unusually long. As a possible explanation for this long-lasting negativity, gender category processes might be tested on many levels and/or in some circles of re-entrant mechanisms. In other word, we suggest that the automatic identification of the gender difference is a fairly complex process,—hence the vMMN activity was extended to 200–500 ms post stimulus interval—especially in case of cropped faces (i.e., without the ears and hair).

As the results of Experiment 2 show, vMMN emerged even if the ISI was longer than ~2000 ms. This finding provided ample evidence that the representation of this facial category survives several seconds. Contrary to the absence of ISI effect on vMMN, P1 and N1/N170 components were sensitive to the ISI, but these relationships were complex. As a function of ISI the latency of these components decreased in both cases. However, P1 amplitude decreased and N1/N170 amplitude increased at longer ISI. The ISI effect on the N1/N170 amplitude is attributed to the refractoriness at short ISI, but at this stage we have no explanation for the other ISI-related differences. As for the N1/N170 component, Mouchetant-Rostaing et al. (2000) demonstrated that the N170 component is insensitive to the processing of genders, and supporting this finding, the present results show gender-related facial processing even in case of compromised N1/N170. However, the contribution of the processes underlying the P1 component in facial processing is a viable possibility (Dering et al., 2011).

At a more general level, the present results provide converging evidence about the automatic development of category-related information, and the automatic detection of events different from the predicted category. Athanasopoulos et al. (2010) obtained larger vMMN in Greek participants for two variants of blue than in British participants. In the Greek language the two variants have different labels, whereas in English only one. Clifford et al. (2010) obtained larger vMMN to between-category colors than to within-category ones, even if the distances in the color space were equal. Finally, Mo et al. (2011) obtained larger within category vMMN if deviants were presented to the right side (i.e., left hemisphere processing). No category-specific difference appeared to left half-field stimulation. VMMN appeared to be sensitive to symmetry as perceptual category (Kecskés-Kovács et al., 2013), hand laterality (Stefanics and Czigler, 2012), and vMMN emerged to deviant facial emotions (Stefanics and Czigler, 2012). The question to be answered in relation to such vMMN results is whether these effects are based on the activation of a common set of physical features, or the stimuli activate the category code, and this code has a top-down effect on stimulus processing. In comparison to other categories, the specificity of the color domain is that the physical stimuli are continuous (visible spectrum) and the categories are products of the perceptual system. Not surprisingly, this characteristic of the color system provided a methodological possibility for investigating language-related effects of vMMN in the studies by Clifford et al. (2010) and Mo et al. (2011); the within and between category stimuli were in equal distance within the color space, therefore such results are difficult to explain without the activation of a category code. Hand laterality is a markedly different category type, it is inherently dichotomous, the distinctive features are relatively simple, and it seems to be impossible to produce continuity between the left and right hand. Gender as perceptual characteristic of facial stimuli seems to be an “immediate” case, the category (female and male) is obviously dichotomous, but on the basis of present results it is challenging to decide whether vMMN was the result of the emergence of the category representation or as an effect of a set of different physical stimulus features. In this study a large set of photographs with different individual features and structural characteristics were presented, therefore the latter possibility is less probable. However, using morphing methods, it is possible to create immediate stimuli. In further studies it would be possible to investigate the sensitivity of vMMN to within category and between category photographs, using similar distance in a morph scale.

In sum, the vMMN components were elicited in both first and second experiments. Deviants elicited a posterior negativity within a comparable latency range in both experiments. The processes of automatic change detection of gender face category are unattached to ISI manipulation, especially as the distributions of vMMNs were similarly enhanced negativities.

In conclusion, in both experiments we found robust vMMN effects, showing that vMMN is sensitive to perceptual categorization processes. Accordingly, emergence of a vMMN response to deviant gender of human faces demonstrates that posterior visual areas automatically registered the unattended gender information, and detected regularities of gender facial stimuli.

### Conflict of interest statement

The authors declare that the research was conducted in the absence of any commercial or financial relationships that could be construed as a potential conflict of interest.

## Footnotes

^1^The term of N1/N170 is an indication that visual stimuli in general elicit a posterior negative exogenous component. However, this negativity is usually larger when the facial stimuli were presented. Therefore, the negative peak can be considered as an aggregate of the N1 component and a putative face-related activity. The equal probability control refers to a method that was developed by Schröger and Wolff (1996). Two aspects are prominent in the control design: 1, the control and deviant stimuli are presented with same features and probabilities; 2, the control sequence is without a sequential regularity rule (Schröger and Wolff, 1996; Jacobsen and Schröger, 2001).

^2^In a control experiment we assessed the gender-related discriminability of the stimuli. It was an active two-stimulus oddball paradigm (active attending) with the infrequent stimuli as target. The sequences were similar to the vMMN sequences, the participants (*n* = 14) were instructed to identify the rare gender category and to respond with button press. We measured RT response and accuracy. Before each block (the block order was randomized) the participants were informed about the current target category (female or male). According to the results, the hit rate was over 80% (mean hit rate of targets = 98.21%, *SD* = 3.61%). Median reaction time was 504.16 ms (*SD* = 65.52 ms). There was no difference between male and female target stimuli. Therefore, we concluded that the two faces were valid and discriminable members of the two categories. However, it must be noted that the experiment design used did not make it possible to examine prototype effect (good and worse members of the categories). Therefore, it is conceivable that if we had used only prototype faces, the vMMN effect would have been more prominent. In a subsequent study by using morphing methods (e.g., 50% female and 50% male or 20% male and 80% female etc.) we could examine the sensitivity of vMMN to within category or between category effects. Finally, to ensure the neutral ecological validity of our stimuli, we ran a control behavioral experiment. It was a three-alternative forced-choice task. The participants (*n* = 14) were instructed to judge emotional expressions of faces on the basis of the following categories: negative facial expression (1st value), neutral facial expression (2nd value) or positive facial expression (3rd value). According to the results, the mean score of male faces was 2.04 (*SD* = 0.53) and the average score of female faces was 2.26 (*SD* = 0.50). We consider the emotional expression of faces were counterbalanced between two face categories i.e., we measured no emotional-related vMMN. Therefore, the difference between the ERPs to the deviant and standard would be a valid gender effect.

^3^Dering et al. (2011) claim that the N170 component is sensitive to cropped face stimuli, whereas the face processes are related to an earlier positive component, to the P1.

## Abbreviations

ANOVA: analysis of variance
ERP: event-related potential
vMMN: visual mismatch negativity
ISI: inter -stimulus -interval.
